# Modeling functional network topology following stroke through graph theory: functional reorganization and motor recovery prediction

**DOI:** 10.1590/1414-431X2022e12036

**Published:** 2022-08-15

**Authors:** S.R.M. Almeida, C.A. Stefano, J. Vicentini, S.L. Novi, R.C. Mesquita, G. Castellano, L.M. Li

**Affiliations:** 1 Departamento de Neurologia, Faculdade de Ciências Médicas, Universidade de Campinas, Campinas, SP, Brasil; 2 BRAINN (Brazilian Institute of Neuroscience and Neurotechnology), Campinas, SP, Brasil; 3 Grupo de Neurofísica, Instituto de Física “Gleb Wataghin”, Universidade de Campinas, Campinas, SP, Brasil

**Keywords:** Betweenness centrality, fMRI, Stroke, Graph metrics, Network analysis

## Abstract

The study of functional reorganization following stroke has been steadily growing supported by advances in neuroimaging techniques, such as functional magnetic resonance imaging (fMRI). Concomitantly, graph theory has been increasingly employed in neuroscience to model the brain's functional connectivity (FC) and to investigate it in a variety of contexts. The aims of this study were: 1) to investigate the reorganization of network topology in the ipsilesional (IL) and contralesional (CL) hemispheres of stroke patients with (motor stroke group) and without (control stroke group) motor impairment, and 2) to predict motor recovery through the relationship between local topological variations of the functional network and increased motor function. We modeled the brain's FC as a graph using fMRI data, and we characterized its interactions with the following graph metrics: degree, clustering coefficient, characteristic path length, and betweenness centrality (BC). For both patient groups, BC yielded the largest variations between the two analyzed time points, especially in the motor stroke group. This group presented significant correlations (P<0.05) between average BC changes and the improvements in upper-extremity Fugl-Meyer (UE-FM) scores at the primary sensorimotor cortex and the supplementary motor area for the CL hemisphere. These regions participate in processes related to the selection, planning, and execution of movement. Generally, higher increases in average BC over these areas were related to larger improvements in UE-FM assessment. Although the sample was small, these results suggest the possibility of using BC as an indication of brain plasticity mechanisms following stroke.

## Introduction

Advances in neuroimaging have enabled the study of the reorganization of brain function, which has been identified as one of the fundamental mechanisms involved in motor recovery following stroke (1,2). Recent neurologic research has emphasized the important role of distributed networks in the brain. Although the brain's structure is changed by focal damage, this change influences the function of distant brain regions (3,4). Focal brain lesions resulting from ischemic stroke may yield selective alterations in functional and structural interconnectivity of other brain circuits that are unrelated to motor function, such as the default mode network (5), executive control network (6), and the white matter language pathways (7).

Graph theory has been introduced as a prospective method for studying functional networks in the central nervous system (for a review, see the paper from Bullmore and Sporns (8)), enabling investigation of the motor network functional reorganization after stroke damage (1,9). This approach, based on the interaction (links or edges) of brain regions (nodes), describes important properties of complex systems by quantifying a network's topology (10). Graph methods can build models of complex networks and characterize connection patterns within the brain from a topological organization perspective through metrics such as degree, clustering coefficient, shortest path length, betweenness centrality, and efficiency, among others (8). In particular, the use of graph models for each brain hemisphere can be helpful for understanding the dynamic reorganization of the brain network after stroke and provide clues to the effects on recovery, although the precise biological mechanisms must still be determined (1).

Network randomization may be a final common outcome following stroke damage, resulting from a compensatory but nonoptimized outgrowth of new connections due to an impaired connection pathway (1). Wang et al. (11) showed that motor execution networks in patients were increasingly random over the course of one year of recovery. New axonal outgrowths may be partly responsible for this randomization, but they may not be the only cause. After ictus, other structural and functional changes may also contribute to the continued randomization of network configuration (11). Lee et al. (1) showed that the topological configuration of the network shifted toward a random network 3 months following ictus. The authors also found a relationship between low characteristic path length (which characterizes high network efficiency) in the ipsilesional hemispheric network just after ictus and better recovery 3 months post-stroke (1). However, the cited studies evaluated only patients with moderate to severe motor deficits, while patients with mild motor impairment or without impairment were excluded.

The aim of this work was to investigate the reorganization of network topology in both ipsilesional (IL) and contralesional (CL) hemispheres during functional recovery in two groups of stroke patients: with and without motor impairment. We also attempted to predict motor recovery from the relationship between local topological variations (more specifically, in the premotor cortex, supplementary motor area, and primary sensorimotor cortex) of the functional network and increased motor function.

## Material and Methods

### Subjects and experimental design

A total of 33 patients who had their first-ever stroke were assessed for eligibility. All stroke patients were treated during the subacute phase at the Emergency Department at the University Hospital of the University of Campinas. They were enrolled in this study within the first month for the first evaluation, and they were subsequently reassessed at 3-4 months after stroke, when they were seen for follow-up at the outpatient stroke clinic at the same institution. The inclusion criteria were: 1) ischemic stroke; 2) patients up to 4 weeks from the onset of ictus; and 3) motor deficits of the contralesional upper and/or lower extremities. The exclusion criteria were: 1) patients whose symptoms completely resolved within 24 h; 2) hemorrhagic strokes or other neurological disorders; and 3) any contraindication for MRI.

**Figure 1:**
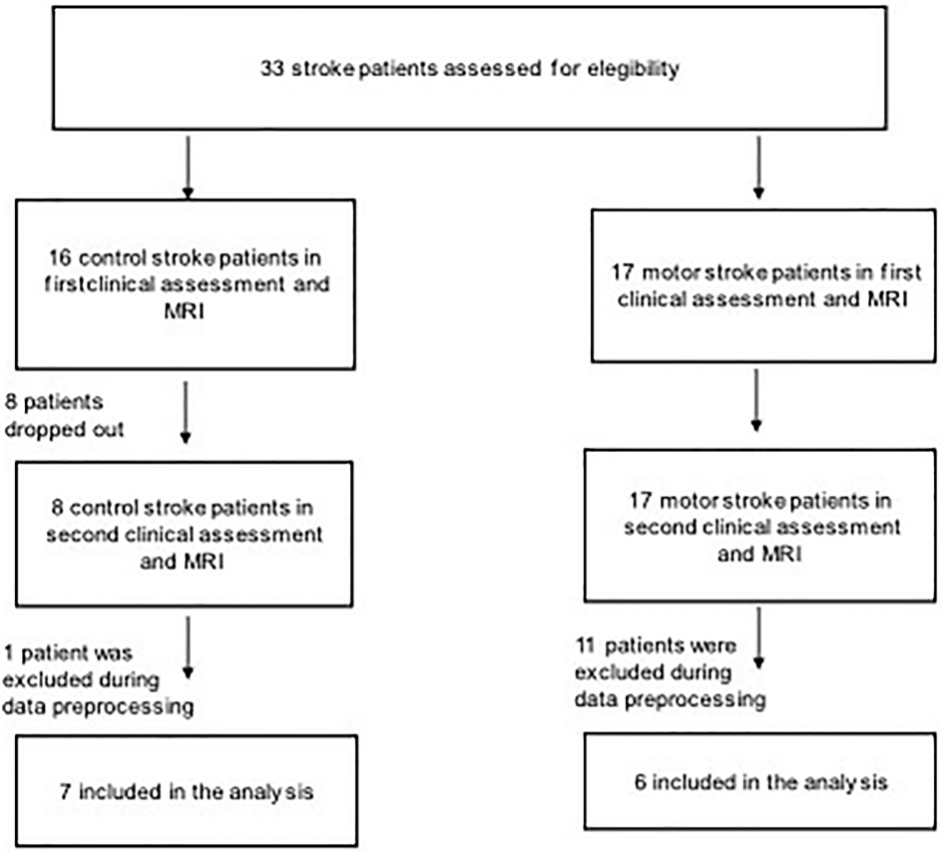
Patient inclusion process for the longitudinal observational study involving two functional magnetic resonance imaging exams, one within 1 month and another 3 months after stroke onset.

Twenty patients out of 33 were excluded, and 13 patients participated in this study (Figure 1). All patients underwent two fMRI scans: one performed within 1 month and another 3 months after stroke onset. Experiments were conducted with the understanding and written consent of each participant, and ethics approval was provided by the University of Campinas Ethics Committee (document number: 694.729; CAAE: 0841814.0.0000.5404).

### Clinical assessment

All patients had stroke and underwent clinical assessment on the same days as the MRI scans. All patients were assessed using the modified Rankin (12) score and the Barthel index (13) to evaluate functionality and daily activities, that is, the ability to carry out everyday tasks. Patients were assigned to one of the two groups depending on their first clinical assessment: with (motor stroke group) or without (control stroke group) motor impairment. Patients without impaired functionality had a Rankin score of 0 or 1 and a Barthel score of 100. Patients with impaired functionality had Rankin scores ≥1 and Barthel scores <100 and were also evaluated using the Fugl-Meyer assessment for motor function of the upper and lower extremities (14). The Fugl-Meyer assessment evaluates sensitivity, speed, coordination, and motor function and is scored between 0 and 2 (0: unable, 1: partly able; 2: fully able to complete movement) with a total score range of 0-66 for upper extremities (UE-FM) and 0-34 for lower extremities (LM-FM) (14). To measure stroke severity, we used the National Institutes of Health Stroke Scale (NIHSS) (15).

### fMRI data acquisition and preprocessing

Patients were resting and awake in the scanner with their eyes closed and were instructed not to think about problems or stressful situations during the exam. The MRI protocol included a 3D-T1 weighted image (isotropic voxels of 1 mm^3^ acquired in the sagittal plane, flip angle=8°, repetition time (TR)=7 ms, echo time (TE)=3.2 ms, matrix=240×240, FOV=240×240×180 mm^3^) and a functional acquisition (echo planar image-EPI; isotropic voxel of 3 mm^3^, 39 slices, no gap, FOV 240×240×117 mm^3^, flip angle=90°, TR=2 s, TE=30 ms, and 180 time points).

We preprocessed the data with the UF^2^C toolbox (16), a MATLAB suite (mathworks.com) for MRI data preprocessing specifically focused on functional connectivity analysis. The preprocessing included functional image realignment, functional-structural images coregistration, and tissues segmentation, normalization, and smoothing. The images origins were manually set at the anterior commissure for all subjects to minimize distortions resulting from the coregistration and normalization steps. In addition, subjects who had more than 30 discarded volumes due to movement constraints were excluded from this study (step known as “scrubbing”, see Power et al. (17) for a review).

Finally, the BOLD time series were pre-whitened following the approach suggested by Santosa et al. (18) to remove the signal autocorrelation. This enables the calculation of Pearson correlation coefficients for estimating the functional networks while avoiding inflated correlation values, yielding more reliable and meaningful results (19) and, hence, a more accurate graph representation of the brain's functional connectivity (FC). All images were flipped to constrain the lesion's location to the right brain hemisphere (i.e., all patients' lesions were constrained to be on the positive MNI x-coordinates by simply inverting the signal of the voxels along the x-axis when the lesion was located on the left hemisphere).

### Data analysis and functional networks

Functional networks were modeled as a graph, a mathematical tool composed of a set of nodes (or vertices), which in this case represented brain regions, and links (or edges), structures that dictate interactions between the nodes. Each node was defined according to the functional atlas proposed by Power et al. (20), and the strength of the links between nodes was calculated using the Pearson correlation, yielding a 264×264 connectivity matrix. Each node was centered at the coordinates suggested by the functional atlas, encompassing all of its nearest neighbors within a three-voxel radius. Hence, each node's time series consisted of the average signal of the aforementioned voxel neighborhood.

To further avoid spurious correlations, following common trends for thresholding the connectivity matrix (21), only the 20% strongest connections were maintained in the graph's adjacency matrix (*A*). This approach also ensured that the graphs of all subjects had the same total number of functional links. Therefore, each entry *a*_*ij*_ of *A* could take the value of either ‘1' or ‘0', indicating whether there was a connection between nodes *i* and *j*; that is:

$$a_{ij}=\left\{\begin{array}{l} 1,\;\;\;\;\;\;\;\;\;\;\text{if}\;i\;\text{and}\;j\;\text{are connected}\\ 0,\;\;\;\;\;\;\;\;\;\;\;\;\;\;\;\;\;\;\;\;\;\;\;\;\;\;\;\;\;\text{otherwise} \end{array}\right.$$ (Eq. 1)

The functional connectivity analysis consisted of assessing how specific metrics varied across fMRI acquisitions for the two patient groups. The chosen metrics were degree, clustering coefficient, characteristic path length, and betweenness centrality. The degree and the clustering coefficient were selected for their easy-to-interpret yet powerful meaning. The characteristic path length was chosen to provide complementary information regarding the efficiency of connections within the network, and the betweenness centrality was selected to provide a notion of the importance of a node in the sense of information flow within the network. A brief description of these metrics is provided below, as they will be needed for interpreting the results. For further details, the interested reader may refer to previous studies (22,23).

The degree for a node *i* (*D_i_*) represents its number of connections, that is (22):

$$D_{i}\;=\sum_{j=1}^{N}a_{ij}$$ (Eq. 2)

where *N* is the total number of nodes in the network (in this case, N=264). The higher the degree of a node, the greater the number of nodes it connects to.

The clustering coefficient (CC) provides an idea of clustering of neighboring nodes: given any three nodes *i*, *j*, and *k*, if *i* and *j* are connected, as well as *j* and *k*, the CC can be thought of as the probability of *i* and *k* also being connected. Mathematically, the CC for a node *i* can be calculated as (22):

$$CC_{i}=\frac{2\sum_{j=1}^{N}\sum_{k=1}^{N}a_{ij}a_{jk}a_{ki}}{k_{i}\left(k_{i}-1\right)}$$ (Eq. 3)

The shortest path length represents the number of links that compose the shortest path between nodes *i* and *j (l)_ij_*. If there is no connection between *i* and *j*, then *l_ij_* = ∞. The characteristic path length of node *i*, then, is simply the average value of *l_ij_*, that is (22):

$$\langle l_{i}\rangle =\frac{1}{N-1}\sum_{j=1}^{N-1}l_{ij}$$ (Eq. 4)

The BC is a metric that describes the importance of a node regarding how significantly it acts as a bridge between two other nodes within the network. For node *i*, this metric can be calculated as (22):

$$BC_{i}=\frac{2}{\left(N-1\right)\left(N-2\right)}\sum_{j\neq i\neq k}\frac{l_{jk}\left(i\right)}{l_{jk}}$$ (Eq. 5)

in which *l_jk_* represents the number of shortest paths that go from *j* to *k*, and *l_ij_* (*i*) represents those that specifically pass through node *i*.

Finally, changes in metrics between the two fMRI scans were calculated as follows:

$$\Delta M\;=\frac{M_{2}-M_{1}}{M_{1}}$$ (Eq. 6)

in which *M* refers to one of the aforementioned metrics, and indices 1 and 2 represent *M*'s values on the first and second scans, respectively. Therefore, *ΔM* indicates a relative value for the change in this metric regarding its magnitude in the first fMRI evaluation.

Metric variations were attributed to standardized anatomical regions of the AAL atlas by comparing its coordinates to those of the functional atlas used in this study. The regions of interest found in our analyses, which are related to sites where *ΔM* variations were more prominent, are reported in Table 1.

**Table 1:** Regions of interest analyzed through anatomical regions of the automated anatomical labeling atlas.

Region	Abbreviation	Side	Node coordinates			Region	Abbreviation	Side	Node coordinates		
			x	y	z				x	y	z
Lentiform nucleus	LN	Left	-22	7	-5	Postcentral gyrus	PoCG	Left	-7	-33	72
		Right	23	10	1				-40	-19	54
Inferior parietal lobule	IPL	Left	-42	-55	45				-29	-43	61
		Right	44	-53	47				-21	-31	61
Parahippocampal girus	PHG	Left	-26	-40	-8			Right	13	-33	75
		Right	27	-37	-13				10	-46	73
Middle frontal gyrus	MFG	Left	-35	20	51				29	-39	59
			-42	38	21				50	-20	42
			-34	55	4				42	-20	55
			-28	52	21	Medial frontal gyrus	MlFG	Left	-3	2	53
			-32	-1	54				-3	44	-9
		Right	34	38	-12				-11	45	8
			19	-8	64				-2	38	36
			23	33	48				-3	26	44
			42	0	47			Right	2	-28	60
			29	-5	54				3	-17	58
Inferior frontal gyrus	IFG	Left	-46	31	-13				7	8	51
		Right	48	22	10				6	64	22
Middle temporal gyrus	MTG	Left	-46	-61	21				8	42	-5
			-58	-26	-15	Superior frontal gyrus	SFG	Left	-18	63	-9
		Right	58	-53	-14				-16	29	53
			51	-29	-4				-10	55	39
Precentral gyrus	PrCG	Left	-45	0	9				-20	64	19
			-55	-9	12				-21	41	-20
		Right	20	-29	60				-39	51	17
			44	-8	57			Right	10	-17	74
			51	-6	32				22	39	39
			56	-5	13				13	55	38
Thalamus	Th	Left	-10	-18	7				13	30	59
		Right	9	-4	6				26	50	27
Insula	In	Left	-38	-33	17	Precuneus	PrCu	Left	-7	-52	61
		Right	32	-26	13				-7	-71	42
			36	22	3				-16	-77	34
Superior temporal gyrus	STG	Left	-51	8	-2			Right	15	-63	26
			-49	-26	5				4	-48	51
			-44	12	-34				10	-62	61
		Right	65	-33	20	Superior parietal lobule	SPL	Left	-17	-59	64
Inferior temporal gyrus	ITG	Left	-56	-45	-24			Right	25	-58	60
			-50	-7	-39	Cingulate gyrus	CG	Left	-14	-18	40
		Right	65	-12	-19				-2	-37	44
			49	-3	-38				-2	-35	31
Cuneus	Cu	Left	-8	-81	7				-1	15	44
			-14	-91	31			Right	8	-48	31
			-3	-81	21				5	23	37
Middle occipital gyrus	MOG	Left	-26	90	3	Claustrum	Cl	Left	-34	3	4
		Right	37	-81	1						
								Right	-	-	-
Inferior occipital gyrus	IOG	Left	-	-	-	Posterior cingulate	PoC	Left	-11	-56	16
									-3	-49	13
		Right	27	-97	-13			Right	11	-54	17
			43	-78	-12						
Lingual gyrus	LG	Left	-25	-98	-12	Fusiform gyrus	FG	Left	-34	-38	-16
			-12	-95	-13						
			-15	-72	-8						
		Right	26	-79	-16			Right	46	-47	-17
Anterior cingulate	AC	Left	-3	42	16			Left	-31	-10	-36
			-11	26	25						
		Right	10	22	27	Uncus	Un	Right	33	-12	-34
			12	36	20						

Finally, the last analysis involved seeking correlations between variations in the average BC and the UE-FM scale on the primary sensorimotor cortex, premotor area, and supplementary motor area.

## Results

Of the 13 participating stroke patients, six presented impaired motor function (motor stroke group, mean age 63±9 years, range 53-80), and seven did not present impaired motor function (control stroke group, mean age 58±9 years, range 49-72). Table 2 presents the baseline characteristics for all subjects. There were no statistically significant differences between the groups regarding age, sex, time post-stroke, or stroke hemisphere. The patients in the motor stroke group had statistically worse scores on the modified Rankin score and Barthel index (see Table 2).

**Table 2:** Demographic and clinical characteristics of the motor stroke and control stroke groups.

Characteristic	Motor stroke group	Control stroke group	P value
Age (mean; min-max)	63 (53-80)	58 (49-72)	0.361^#^
Gender (n, %)			1.00*
Male	4 (66.7)	5 (71.4)	
Female	2 (33.3)	2 (28.6)	
Time after stroke (mean; days)	25	23	0.604^#^
Stroke hemisphere (n, %)			0.592*
Right	3 (50.0)	5 (71.4)	
Left	3 (50.0)	2 (28.6)	
NIHSS (mean; min-max)	2.8 (0-8)		
UE-FM (mean; min-max)	62 (50-66)		
LE-FM (mean; min-max)	32.3 (30-34)		
Barthel (mean; min-max)	90 (85-95)	100	0.001^+^
Rankin index (n, %)			0.003^#^
0		4 (57.1)	
1	2 (33.3)	3 (42.9)	
2	4 (66.7)		
3			
4			
5			

UF-FM: upper-extremity Fugl-Meyer assessment; LE-FM: lower-extremity Fugl-Meyer assessment; ^#^ *t*-test; ^+^Mann-Whitney U test; *chi-squared test (significant values at P<0.05).

### Group average results

**Figure 2:**
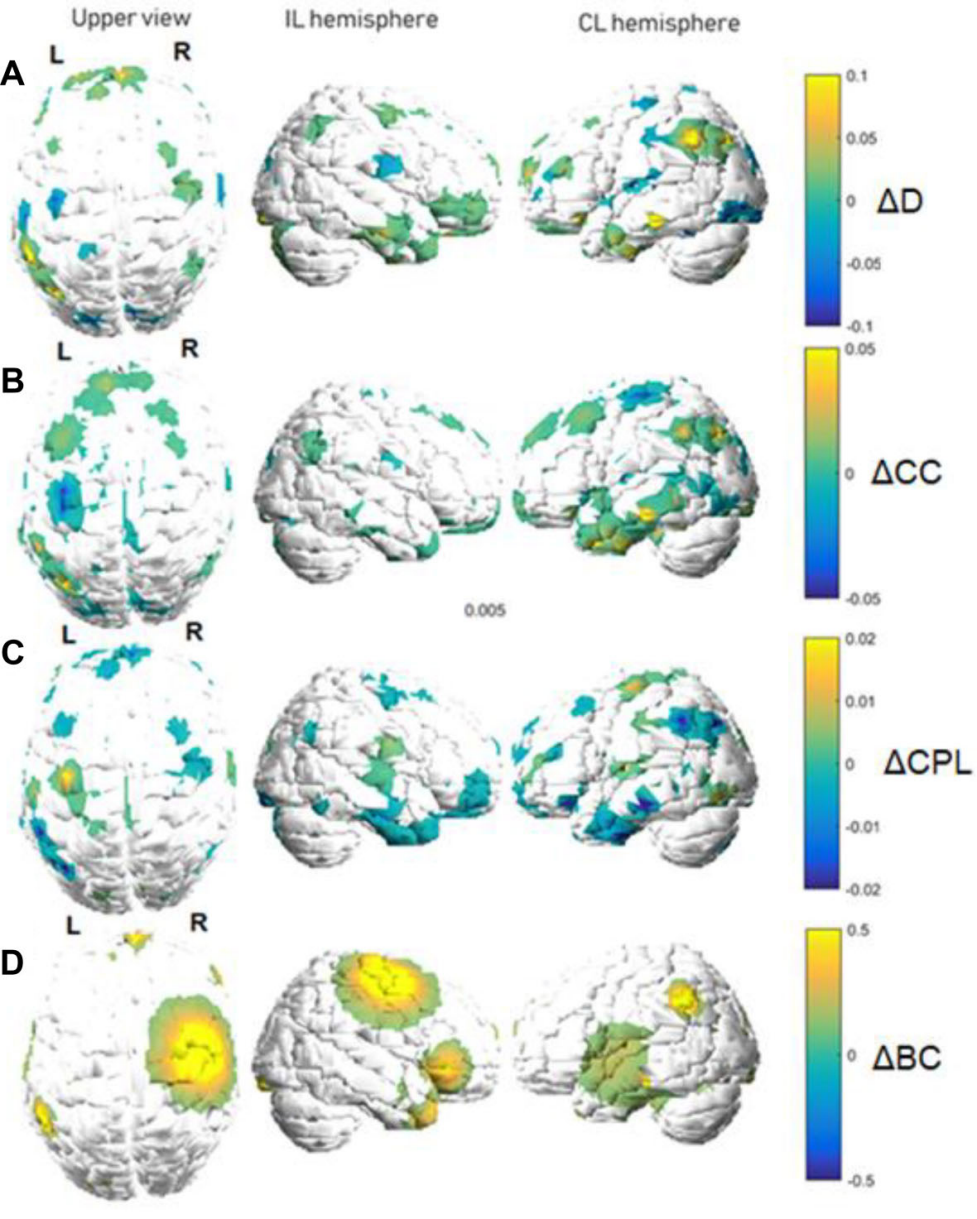
Graph metric changes between functional magnetic resonance imaging acquisitions (motor stroke group). Variations are shown in a relative color scale, according to Equation 6. **A**, degree (D); **B**, clustering coefficient (CC); **C**, characteristic path length (CPL); **D**, betweenness centrality (BC). IL: ipsilesional; CL: contralesional.

**Figure 3:**
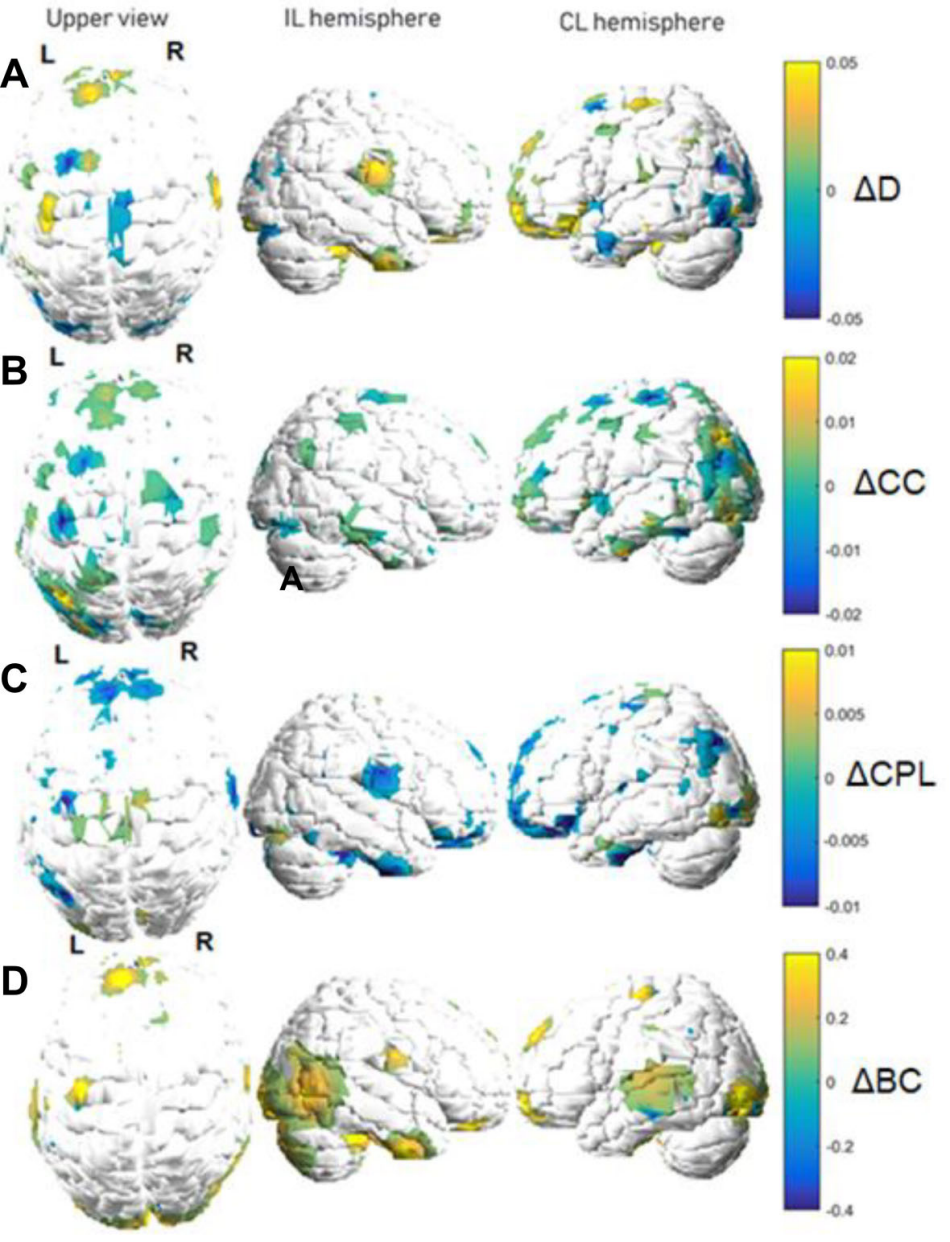
Graph metric changes between functional magnetic resonance imaging acquisitions (control stroke group). Variations are shown in a relative color scale, according to Equation 6. **A**, degree (D); **B**, clustering coefficient (CC); **C**, characteristic path length (CPL); **D**, betweenness centrality (BC). IL: ipsilesional; CL: contralesional.

Graphs of metric variations are displayed in color maps and in relative (percentage) scales. Each metric will be discussed separately, as each one carries a specific and unique meaning regarding its interpretation within the functional network. Figures 2 and 3 show variation maps for the studied metric between both fMRI acquisitions for patients of the motor stroke group and control stroke group, respectively. The behavior shown in the figures was estimated by taking the average connectivity matrix for each patient group. Areas closer to yellow indicate increases, while those closer to blue indicate decreases. In these maps, the white areas indicate values considered too small to be displayed, that is, the white regions in Figures 2 and 3 indicate unplotted values. Only variations that exceeded half the average *ΔM* across the whole brain are shown. Finally, we reiterate that the images of some patients were flipped to restrict the lesions to the right hemisphere in all subjects.

To further investigate the patterns of the regions in Figures 2 and 3, we computed average values for the metrics of the cortical and subcortical areas with the largest variations. The results are shown in Table 3, with motor stroke and control stroke groups separated by graph metric and hemispheres, as well as for increase or decrease of the corresponding metric, which are indicated as upwards and downwards arrows, respectively. Note that some regions presented both increases and decreases, and thus both arrows are displayed. We interpreted the presence of both increasing and decreasing trends as changes belonging to distinct portions (i.e., distinct graph nodes) of the same anatomical region.

**Table 3:** Increase (↑) or decrease (↓) of graph metrics in the motor stroke and control stroke groups.

Graph metric	Control stroke group		Motor stroke group	
	CL	IL	CL	IL
D	LN (↑,↓)	LN (↓)	LN (↑)	MFG (↑,↓)
	STG (↑,↓)	MFG (↑)	IPL (↑)	IFG (↑)
	SFG (↑)	PrCG (↑,↓)	PHG (↑)	MTG (↑)
	IFG (↑)	PoCG (↑)	STG (↓)	PrG (↑)
	Cl (↑)	IOG (↓)		ITG (↓)
	PrCu (↑,↓)	Th (↑)		Th (↑,↓)
	IPL (↓)	PrCU (↓)		In (↑)
CC	PHG (↑,↓)	CG (↑,↓)	LN (↓)	MFG (↓)
	SFG (↑,↓)	LG (↑)	MlFG (↓)	MTG (↓)
	STG (↑)	FG (↑)	SFG (↓)	PrG (↑)
	PoC (↑)	IFG (↓)	STG (↑)	PoCG (↓)
	PoCG (↓)	MFG (↑)	ITG (↑,↓)	MlFG (↑)
		MTG (↓)	SPL (↓)	
		PrCG (↑,↓)	PoCG (↑)	
		Th (↑)	In (↑)	
		Cu (↑)	PrCu (↓)	
CPL	PHG (↑)	MFG (↓)	PHG (↑)	MFG (↑)
	PrCG (↑,↓)	PHG (↑)	STG (↑,↓)	MlFG (↓)
	PoCG (↓)	FG (↑,↓)	PoCG (↑)	PrCG (↑,↓)
	STG (↑,↓)	PrCG (↓)	MOG (↓)	CG (↑)
	ITG (↑)	PoCG (↑,↓)	In (↑)	Cu (↑)
	SFG (↑,↓)	LG (↑)	IPL (↓)	
	In (↑)	CG (↓)		
		Th (↑)		
BC	LN (↑,↓)	LN (↑)	LN (↑,↓)	MFG (↑,↓)
	SFG (↑,↓)	MTG (↑)	IPL (↑)	IFG (↑)
	MFG (↑)	STG (↑,↓)	SFG (↑)	PRrCG (↑,↓)
	MlFG (↑,↓)	PrCG (↑)	STG (↑)	PoCG (↑,↓)
	PoCG (↑)	LG (↑,↓)	ITG (↑)	Th (↑)
	STG (↑,↓)	IFG (↑)	CL (↓)	
	ITG (↓)	Un (↑)		
	AC (↑)	IPL (↑)		
	In (↑)	Cu (↑,↓)		
	PrCu (↑)			
	IPL (↓)			

CL: contralesional; IL: ipsilesional; D; degree; CC: clustering coefficient; CPL: characteristic path length; BC: betweenness centrality; For region abbreviations, see Table 1.

### Individual BC results

**Figure 4:**
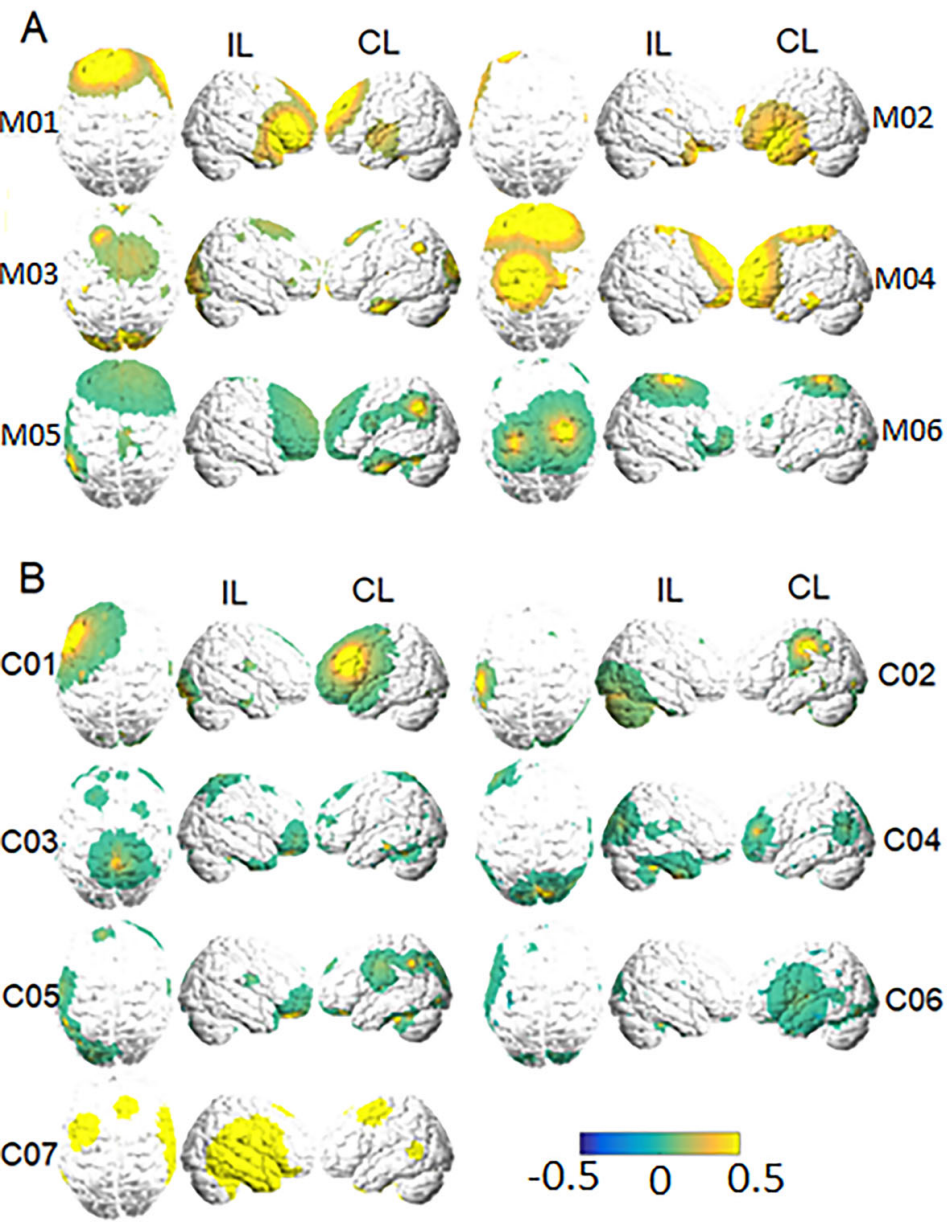
Individual betweenness centrality (BC) variation plots for the groups with (**A**) and without (**B**) motor-impairment. ‘IL' and ‘CL' indicate ‘ipsilesional' (the right hemisphere) and ‘contralesional' (the left hemisphere), respectively. Variations are shown in a relative color scale, according to Equation 6. Subjects from the motor stroke and control groups are labeled with ‘M' and ‘C', respectively.

Based on the results in Figures 2 and 3 indicating that BC was the metric with the largest changes in fMRI acquisitions, we further explored its variations for each subject. These results are shown in Figure 4 for all participants in both the motor impairment (A) and control stroke (B) groups.

**Figure 5:**
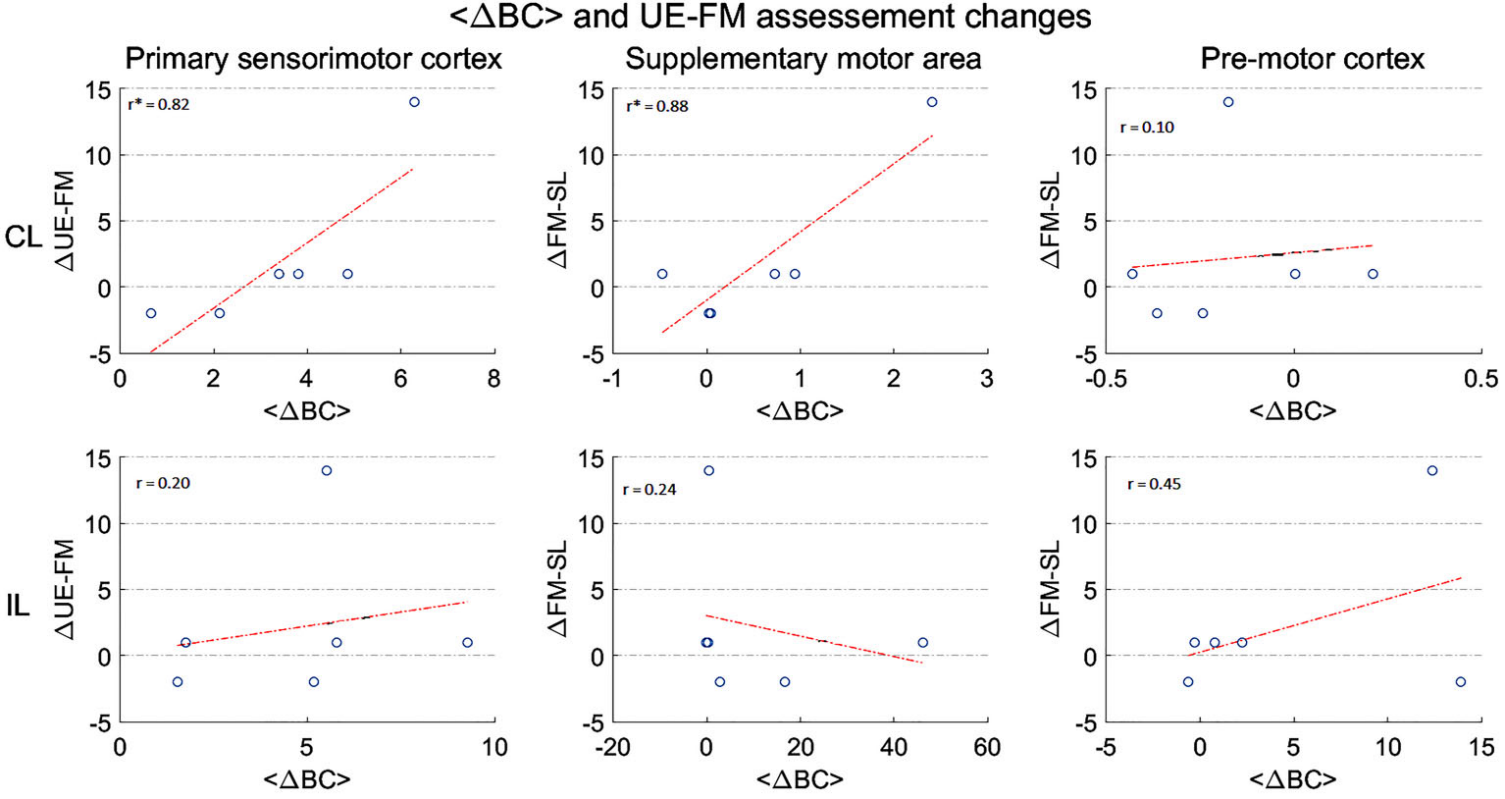
Relationship between upper-extremity Fugl-Meyer (UE-FM) scale changes and average betweenness centrality (BC) variation for the motor stroke group. In this plot, each point corresponds to the <ΔBC> and ΔUE-FM values of a subject. Plots in the upper and lower panels correspond to the contralesional (CL) and ipsilesional (IL) hemispheres, respectively. Each column designates <ΔBC> values gathered at specific areas. The correlation strength between the two variables, i.e., the <ΔBC> and the corresponding changes in UE-FM (indicated by ΔUE-FM), are shown as r, that indicates the value of the correlation coefficient. When the P corresponding to that rho is less than 0.05, the correlation is significant. The correlation strength between the two variables is shown with an asterisk (*) when significant (P<0.05).

In addition, we also investigated whether this metric correlated with the UE-FM by studying how the average BC change for a given subject was related to the Fugl-Meyer score change (Figure 5). For this analysis, three areas of interest were explored: the primary sensorimotor cortex, the supplementary motor area, and the premotor cortex for both the CL (upper panels) and IL (lower panels) hemispheres. The correlation strength between the two variables, i.e., the <ΔBC> and the corresponding changes in UE-FM (indicated by ΔUE-FM), are shown as r, that indicates the value of the correlation coefficient. When the p corresponding to that rho is less than 0.05, the correlation is significant, with an asterisk indicating statistical significance (P<0.05).

## Discussion

### Motor stroke group

#### Group average results

Several studies have reported alterations in brain topological organization and disruptions in functional connections in patients following stroke when compared to healthy controls. As expected, there is a decrease in global efficiency, indicating a reduced capacity for information transfer across the entire stroke brain (9,24). In this work, to better understand brain changes after stroke, we compared functional network changes between groups of stroke patients with different stroke outcomes, namely, with and without motor impairment. Interestingly, robust changes in global integration, including alterations in strength, clustering coefficient, characteristic path length, and betweenness centrality, were identified in both groups, as well as a correlation of these network changes with clinical variables that assess motor impairment (for the motor stroke group).

As widespread brain regions and extensive networks may be damaged in stroke patients, studies investigating the whole network of functionally interacting brain regions may be more valuable for a better understanding of the pathological mechanisms of stroke than studies investigating local connections (9). In this context, we found several connectivity alterations over time, not only in the lesion area but also in the frontal and temporal regions, the parietal gyrus, and the basal ganglia, in both hemispheres. Although it is tempting to attribute these changes to brain reorganization as a mechanism to suppress further impairments due to the stroke, it is important to be cautious with these conjectures, given that the analyzed patient sample was small, and the patients had a great variability regarding the damaged brain areas.

The largest increases in metrics on the IL hemisphere (Figure 2A) were observed in the frontal and temporal areas. In the CL hemisphere, the largest increases were observed in the parietal cortex (Brodmann 40), and the largest decreases occurred in the superior temporal cortex. Some gyri were common to both hemispheres, presenting the highest variations (Table 3), but the degree tended to increase more prominently in the CL hemisphere. In other words, the number of connections tended to increase more significantly contralateral to the lesion. An increased degree (i.e., increase in the number of functional connections) for specific regions implies that such regions display more synchronicity of their recorded fMRI time series at a global level (that is, considering every other region of interest in the brain).

For the clustering coefficient (Figure 2B), the CL hemisphere tended to present the most significant cortical increases, especially in areas such as the superior and inferior temporal gyri and the postcentral gyrus. Ipsilateral to the lesion, most displayed locations remained at approximately the same values for this metric, with the largest (yet mild) clustering coefficient increases being observed on the precentral and medial frontal gyri (Table 3). In addition, altered areas for the CC were similar to those involving the degree (Figure 2A and B); relative variations, however, were greater for the latter metric (CC highest changes were approximately 5%, while maximum degree changes were approximately 10%). Since the CC considers functional interactions at a more local scale, as it accounts for elements of the adjacency matrix that are nearest neighbors to each another [see Equation 3], when analyzed simultaneously with degree, it can further indicate whether, to any extent, the variations in the number of functional connections of a given region of interest were spatially restricted to it. In this case, a qualitative visual inspection of Figure 2A and B suggests that this seemed to be the case for the motor stroke group.

The characteristic path length (Figure 2C) followed the same principle as the other metrics, with increased values in the CL hemisphere. Increases in the CPL imply that the information must travel through a larger number of functional links from origin to destination. Hence, regions with increased (or decreased) CPLs can be regarded as requiring a higher (or smaller) number of functional connections to transfer or receive information, indicating a loss (or gain) of information transfer efficiency.

To summarize, when concomitantly analyzing the findings for the three aforementioned metrics, the CL (or IL) increased (or decreased) for the degree, and the CC indicated a higher (or lower) number of connections and efficiency in local communication of a node, resulting in a smaller (or larger) CPL. In other words, there seems to be a tendency for the CL hemisphere to become more efficient in information transfer, perhaps due to compensatory mechanisms following a stroke in the other hemisphere.

Greater connectivity and efficiency of the CL hemisphere may occur to compensate for loss of connectivity in the ischemic region. Functional neuroimaging studies suggest that activity in the sensorimotor network or ipsilesional motor cortex, is most abnormal in the early phase after hemiparetic stroke, and that motor recovery is related to normalization of activity (25). In a previous cross-sectional study, we had already demonstrated increased connectivity on the CL hemisphere in stroke patients with impaired functionality (6). It is possible that the normalization of brain activity occurs later. Lee et al. (1) suggest that the damaged brain enters a chronic stage and further reorganization might not occur because most network reorganization usually happens during the first 3 months after stroke.

Finally, the BC (Figure 2D) displayed the most dramatic variations, with increases of more than 50%. Additionally, no significant decreases were observed. The regions with high values of BC are considered network hubs, and group differences in BC of nodes reflect effects of the disease on the global roles of regions in the network (26,27). Similar to the works by Zhang et al. (9) and Yin et al. (24), we verified an increased nodal BC in the CL superior frontal gyri (identified as supplementary motor area) and the CL inferior parietal lobule, which may participate in processes related to movement selection and movement planning. These areas may be viewed as reflecting the selective neuroplastic recruitment of the unaffected motor network to compensate for the damage induced by the lesion. Furthermore, increases occurred in subcortical areas, such as the basal ganglia and thalamus, structures involved in known motor circuits.

#### Individual BC results

When analyzing BC variations individually, it is noticeable how, despite existing common trends among all subjects, there were many particularities. However, overall, frontal, and parietal areas were common sites of the largest alterations (although the spatial extent of the involved sites is very subject-dependent). BC can be an important parameter to identify changes in functional reorganization since it increased in the frontal and parietal regions, which are responsible for motor planning and integration of sensory input and motor output. This reflects the clinical evolution of patients with motor deficits over time, since there was an improvement in functionality and greater independence in daily activities, shown by the Rankin and Barthel scales and by the Fugl-Meyer assessment. However, the sample was too small for further inferences about statistical significance.

Furthermore, the results in Figure 5 showed that the average changes in the BC and UE-FM assessments were significantly correlated for the primary sensorimotor cortex and the supplementary motor area for the CL hemisphere. On the other hand, no significant correlation was found for the same areas on the affected hemisphere, nor for both hemispheres for the premotor cortex. This means that both the increase in importance of the primary sensorimotor area and the supplementary motor area regarding information flow through the brain - as reflected by the BC metric - were actually linked to clinical improvement of patients over time. This finding suggested that the BC in these areas might be an indicator of neural plasticity related to motor recovery. Moreover, the fact that these changes were located on the CL hemisphere may suggest some type of compensatory mechanism by the unaffected areas. Nevertheless, further analyses are still necessary at this point, as our small number of subjects limits the generalizability of our results.

### Control stroke group

#### Group average results

Most change patterns were similar to those found for the motor-impaired patients. The degree and clustering coefficient presented the most significant increases in the CL hemisphere. As with the motor impairment patients, most sites with increased degree (such as the frontal and temporal gyrus) also roughly exhibited larger clustering coefficients. The metric's changes were restricted to a small range, from 2 to 5% (Figure 3A and B).

As observed for the motor stroke group, these patients displayed decreases (or increases) in the CPL in the proximities of sites that exhibited increases (or decreases) in the degree and CC (Figure 3C). However, locations encompassing structures such as the superior frontal, superior temporal, middle frontal, precentral, and postcentral gyri showed considerable decreases (although these variations were small compared to the other metric, of approximately 1%).

The BC alterations were also the most considerable in this group, reaching values up to 40%. The main areas of increase in the CL included the superior frontal, superior temporal, and inferior temporal gyri, whereas IL increases mainly occurred in the middle frontal, inferior frontal, precentral, and postcentral gyri. Unlike the motor-impaired patients, the average for the control group also displayed considerable BC increases in the proximities of the cerebellum (Figure 3D).

Indeed, several studies have shown an increase in cerebellar activity in stroke patients compared with healthy individuals (11,24). In the present study, patients without motor deficits also showed increased cerebellar connectivity over time. Most likely, the greater motor performance of these patients compared with patients with motor deficits may have been influenced by activation of the cerebellum, since this region interferes with coordination and motor learning.

In a previous work, we found higher connectivity in some non-motor networks in healthy controls than in stroke patients with impaired functionality (6). Now, patients in the control group had more regions with variations in connectivity over time than the motor deficit group, either increasing or decreasing. One hypothesis is that these modifications and the alterations of other areas occurred because it is not only the motor circuit that can help restore the compromised side. However, it is still unclear how all these changes, even with reduced connectivity, can interfere with the process of brain reorganization and functional recovery.

#### Individual BC results

Overall, patients in the control stroke group presented a tendency to display the most significant increases in the CL hemisphere (Figure 4B; see, e.g., Subjects 1, 2, 3, 4, and 5). Moreover, occipital areas and sites close to the cerebellum were more prominent regarding BC changes for this group than for the motor stroke group (Figure 4B). Subject 7, in particular, presented the highest and most noticeable variations.

Although some studies indicate that patients with good functional rehabilitation outcome usually have activation in the perilesional area during functional activities (28), other findings indicate CL activation, which is more in agreement with our present findings. Indeed, Calautti et al. (29), Fujii et al. (30), and Almeida et al. (6) suggest that increased activity in CL sensorimotor cortices is an available mechanism for compensating, at least partially, for stroke-induced motor impairments. Also, Xu et al. (31) and Swayne et al. (32) reported that while it is unclear how the CL activity may influence motor recovery, the reorganization of residual tissue to re-enable motor function likely depends on some degree of intracortical disinhibition to allow access to additional networks.

### Conclusions

In this study, we investigated how graph metrics vary over time between two distinct fMRI acquisitions for patients with different types of impairment following stroke. We found that most metrics displayed only slight changes in all patients with stroke, with the exception of betweenness centrality, which showed changes of up to 50% for motor-impaired patients and 40% for control patients.

On further investigation of BC alterations, we found significant correlations between average change in BC and alterations in the UE-FM score for the CL hemisphere in the primary sensorimotor cortex and in the supplementary motor area for the motor impaired group, up to 3-4 months after stroke. However, additional investigations with a larger number of subjects are necessary to verify whether this relationship can be generalized. Therefore, at this point, we can only speculate that BC may reflect aspects underlying brain plasticity mechanisms after stroke. These results should be explored in future studies in the field.

## References

[1] Lee J, Lee M, Kim DS, Kim YH (2015). Functional reorganization and prediction of motor recovery after
a stroke: a graph theoretical analysis of functional
networks. Restor Neurol Neurosci.

[2] Cheng L, Wu Z, Fu Y, Miao F, Sun J, Tong S (2012). Reorganization of functional brain networks during the recovery
of stroke: a functional MRI study. Annu Int Conf IEEE Eng Med Biol Soc.

[3] Carter AR, Astafiev SV, Lang CE, Connor LT, Rengachary J, Strube MJ (2010). Resting interhemispheric functional magnetic resonance imaging
connectivity predicts performance after stroke. Ann Neurol.

[4] Honey CJ, Sporns O (2008). Dynamical consequences of lesions in cortical
networks. Hum Brain Mapp.

[5] Vicentini JE, Weiler M, Almeida SRM, de Campos BM, Valler L, Li LM (2017). Depression and anxiety symptoms are associated to disruption of
default mode network in subacute ischemic stroke. Brain Imaging Behav.

[6] Almeida SRM, Vicentini J, Bonilha L, De Campos BM, Casseb RF, Min LL (2017). Brain connectivity and functional recovery in patients with
ischemic stroke. J Neuroimaging.

[7] Bonilha L, Nesland T, Rorden C, Fillmore P, Ratnayake RP, Fridriksson J (2014). Mapping remote subcortical ramifications of injury after ischemic
strokes. Behav Neurol.

[8] Bullmore E, Sporns O (2009). Complex brain networks: graph theoretical analysis of structural
and functional systems. Nat Rev Neurosci.

[9] Zhang J, Zhang Y, Wang L, Sang L, Yang J, Yan R (2017). Disrupted structural and functional connectivity networks in
ischemic stroke patients. Neuroscience.

[10] Boccaletti S, Latora V, Moreno Y, Chavez M (2005). Complex networks: structure and dynamics. Phys Rep.

[11] Wang L, Yu C, Chen H, Qin W, He Y, Fan F (2010). Dynamic functional reorganization of the motor execution network
after stroke. Brain.

[12] Rankin J (1957). Cerebral vascular accidents in patients over the age of 60: iii.
Diagnosis and treatment. Scott Med J.

[13] Shah S, Vanclay F, Cooper B (1989). Improving the sensitivity of the Barthel Index for stroke
rehabilitation. J Clin Epidemiol.

[14] Fugl Meyer AR, Jääskö L, Leyman I, Olsson S, Steglind S (1975). The post-stroke hemiplegic patient.1. a method for evaluation of
physical performance. Scand J Rehabil Med.

[15] Brott T, Adams HP, Olinger CP, Marler JR, Barsan WG, Biller J (1989). Measurements of acute cerebral infarction: a clinical examination
scale. Stroke.

[16] de Campos BM, Coan AC, Lin Yasuda C, Casseb RF, Cendes F (2016). Large-scale brain networks are distinctly affected in right and
left mesial temporal lobe epilepsy. Hum Brain Mapp.

[17] Power JD, Schlaggar BL, Petersen SE (2015). Recent progress and outstanding issues in motion correction in
resting state fMRI. Neuroimage.

[18] Santosa H, Aarabi A, Perlman SB, Huppert TJ (2017). Characterization and correction of the false-discovery rates in
resting state connectivity using functional near-infrared
spectroscopy. J Biomed Opt.

[19] Woolrich MW, Ripley BD, Brady M, Smith SM (2001). Temporal autocorrelation in univariate linear modeling of FMRI
data. Neuroimage.

[20] Power JD, Cohen AL, Nelson SM, Wig GS, Barnes KA, Church JA (2011). Functional network organization of the human
brain. Neuron.

[21] van den Heuvel MP, de Lange SC, Zalesky A, Seguin C, Yeo BTT, Schmidt R (2017). Proportional thresholding in resting-state fMRI functional
connectivity networks and consequences for patient-control connectome
studies: issues and recommendations. Neuroimage.

[22] Fornito A, Zalesky A, Bullmore ET (2016). Fundamentals of brain network analysis. Elsevier.

[23] De Vico Fallani F, Richiardi J, Chavez M, Achard S (2014). Graph analysis of functional brain networks: practical issues in
translational neuroscience. Philos Trans R Soc B Biol Sci.

[24] Yin D, Song F, Xu D, Sun L, Men W, Zang L (2014). Altered topological properties of the cortical motor-related
network in patients with subcortical stroke revealed by graph theoretical
analysis. Hum Brain Mapp.

[25] Schaechter JD (2004). Motor rehabilitation and brain plasticity after hemiparetic
stroke. Prog Neurobiol.

[26] He Y, Chen Z, Evans A (2008). Structural insights into aberrant topological patterns of
large-scale cortical networks in Alzheimer's disease. J Neurosci.

[27] Rubinov M, Sporns O (2010). Complex network measures of brain connectivity: Uses and
interpretations. Neuroimage.

[28] Dong Y, Winstein CJ, Albistegui-DuBois R, Dobkin BH (2007). Evolution of fMRI activation in the perilesional primary motor
cortex and cerebellum with rehabilitation training-related motor gains after
stroke: a pilot study. Neurorehabil Neural Repair.

[29] Calautti C, Leroy F, Guincestre JY, Baron JC (2001). Dynamics of motor network overactivation after striatocapsular
stroke: a longitudinal PET study using a fixed-performance
paradigm. Stroke.

[30] Fujii Y, Nakada T (2003). Cortical reorganization in patients with subcortical hemiparesis:
neural mechanisms of functional recovery and prognostic
implication. J. Neurosurg.

[31] Xu H, Qin W, Chen H, Jiang L, Li K, Yu C (2014). Contribution of the resting-state functional connectivity of the
contralesional primary sensorimotor cortex to motor recovery after
subcortical stroke. PLoS One.

[32] Swayne OBC, Rothwell JC, Ward NS, Greenwood RJ (2008). Stages of motor output reorganization after hemispheric stroke
suggested by longitudinal studies of cortical physiology. Cereb Cortex.

